# Regenerative Intestinal Stem Cells Induced by Acute and Chronic Injury: The Saving Grace of the Epithelium?

**DOI:** 10.3389/fcell.2020.583919

**Published:** 2020-11-12

**Authors:** William D. Rees, Rene Tandun, Enoch Yau, Nicholas C. Zachos, Theodore S. Steiner

**Affiliations:** ^1^Department of Medicine, University of British Columbia, Vancouver, BC, Canada; ^2^BC Children’s Hospital Research Institute, Vancouver, BC, Canada; ^3^Division of Gastroenterology and Hepatology, Department of Medicine, Johns Hopkins University School of Medicine, Baltimore, MD, United States

**Keywords:** intestinal epithelium, inflammatory bowel disease, enteroids, Wnt, intestinal stem cells (ISCs)

## Abstract

The intestinal epithelium is replenished every 3–4 days through an orderly process that maintains important secretory and absorptive functions while preserving a continuous mucosal barrier. Intestinal epithelial cells (IECs) derive from a stable population of intestinal stem cells (ISCs) that reside in the basal crypts. When intestinal injury reaches the crypts and damages IECs, a mechanism to replace them is needed. Recent research has highlighted the existence of distinct populations of acute and chronic damage-associated ISCs and their roles in maintaining homeostasis in several intestinal perturbation models. What remains unknown is how the damage-associated regenerative ISC population functions in the setting of chronic inflammation, as opposed to acute injury. What long-term consequences result from persistent inflammation and other cellular insults to the ISC niche? What particular “regenerative” cell types provide the most efficacious restorative properties? Which differentiated IECs maintain the ability to de-differentiate and restore the ISC niche? This review will cover the latest research on damage-associated regenerative ISCs and epigenetic factors that determine ISC fate, as well as provide opinions on future studies that need to be undertaken to understand the repercussions of the emergence of these cells, their contribution to relapses in inflammatory bowel disease, and their potential use in therapeutics for chronic intestinal diseases.

## Introduction

The intestinal epithelium is composed of a variety of intestinal epithelial cells (IECs), each having specific contributions to maintaining normal intestinal function. In addition to critical roles in nutrient absorption and fluid secretion, IECs also provide structural support and a chemical and physical barrier that protects the cells of the underlying lamina propria (LP) from food antigens, commensal microbes, and other cellular insults. In order to maintain this barrier function, IECs undergo constant self-renewal, replenished from a self-sustaining intestinal stem cell (ISC) niche every 3–4 days (Reynolds et al., 2014). It is important that the signaling pathways maintaining this normal turnover remain uncompromised, efficient, and resilient in order to maintain homeostasis.

The IEC renewal cycle can be perturbed by injury, and the epithelium needs mechanisms to repair itself when this occurs. There are a number of ways that a normal intestinal epithelium can be injured, including infection, radiation, ischemia, physical trauma, or immune-mediated injury (e.g., graft-vs.-host disease). If injury is limited to the superficial, differentiated compartment, ISCs can readily repair the damage through their normal regenerative cycling. However, in severe or transmural injury, ISCs can be lost (Tao et al., 2017) and must be replenished with other stem cells that migrate to the site of injury, ultimately helping aid in the maintenance and restoration of the damaged epithelium (Feil et al., 1989; Seno et al., 2009; Miyoshi et al., 2012).

It is essential that these regenerative stem cells successfully replace their damaged counterparts and have similar efficiency in providing barrier integrity and protection against the luminal contents and other cellular insults. While these damage-associated regenerative stem cells (DARSCs) are critical to resolve acute injury, it is not known if they are beneficial or harmful in the setting of chronic intestinal inflammation, such as in inflammatory bowel disease (IBD). Recent studies have demonstrated altered gene expression and innate immune responses in primary enteroids derived from patients with IBD (Kuwabara et al., 2018; Martin et al., 2019; Smillie et al., 2019; Rees et al., 2020b), suggesting the likelihood of long-lasting changes in ISCs. Could this be the result of chronic inflammation in the ISC environment? Moreover, do these chronically damaged ISCs respond to inflammatory signals (which have been demonstrated to maintain homeostasis and affect lineage commitment in the IEC niche) similar to their parental cells? Do DARSCs accumulate epigenetic changes that ultimately contribute to the chronic, relapsing nature of IBD? To address these important questions, we will be highlighting recent literature that has characterized damage-associated ISCs, as well as the signaling factors that drive maturation of ISCs, and examine the potential exploitation of these cells using autologous organoid transplantation as a potential therapeutic tool for diseases of the intestinal epithelium.

## Intestinal Epithelial Cells—The Conveyor Belt That Runs the Gut

The intestinal epithelium is composed of a single cell layer of columnar cells that form the luminal surface of both the small and large intestine. The large intestine (colon), has a relatively flat luminal surface with crypts or glands that penetrate away from the lumen. It is covered by two distinct mucus layers that reduce direct contact with food antigens, commensal microbes, and other molecules that may invoke an immune response (Johansson et al., 2011). The small intestine (SI), in contrast, contains villi that protrude into the lumen to increase the surface area for digestion of food, as well as movement of nutrients, and has one layer of mucus, which also protects against cellular insults.

**FIGURE 1:**
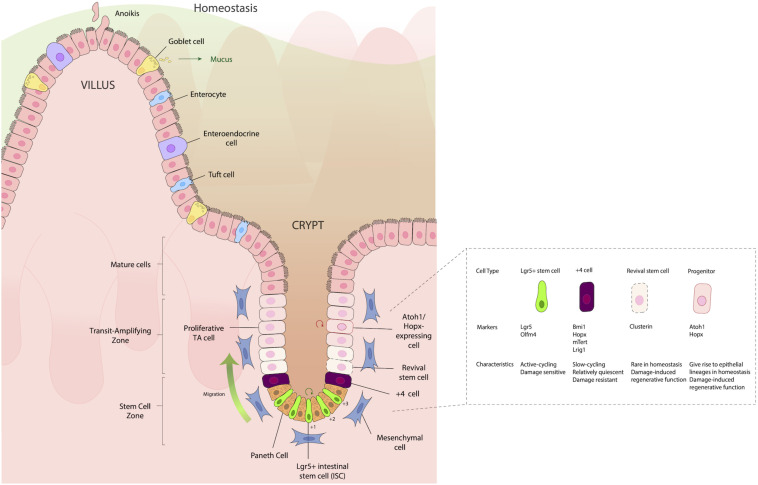
Structure of the intestinal epithelium. The intestinal epithelium is replenished every 3–4 days by rapidly dividing Lgr5^+^ intestinal stem cells (ISCs) in the crypt base that differentiate as they move up the crypt villus axis, until they eventually slough into the lumen. Situated between the small intestinal ISCs are Paneth cells, which are important for providing signaling molecules such as WNT, epidermal growth factor (EGF) and Notch ligands (DLL1, DLL4), which aid in the maturation and differentiation of ISCs to either absorptive (enterocytes), secretory [Goblet cell, enteroendocrine (EE)], or Tuft cell lineages. Just above the IECs, frequently in the “+4” position, are slow cycling, damage resistant cells that express the markers Bmi1, Hopx, mTert, and Lrig1. Located in the transit amplifying zone are Atoh1 and Hopx-expressing mature cell precursors, which give rise to different epithelial lineages during homeostasis, but during damage, have a regenerative function, helping aid in re-establishing damaged epithelia.

The intestinal epithelium can be thought of as a conveyor belt, where cells originate from dividing ISCs at the crypt base and differentiate as they move up the crypt/villus axis, before being sloughed into the lumen. This process takes on average 3–4 days. Situated between ISCs in the SI are Paneth cells, which, unlike other IECs, move downward toward the ISC niche as they differentiate, and provide important signaling molecules such as WNT, epidermal growth factor (EGF), and Notch ligands (DLL1, DLL4) to aid in the maturation of ISCs to absorptive and secretory cell lineages (reviewed in Gehart and Clevers, 2019). In the colon, which largely lacks Paneth cells, mesenchymal cells such as fibroblasts and myofibroblasts instead provide these factors. These signaling molecules can push the ISC progeny to two types of progenitor cells: (1) Secretory progenitors, which can differentiate into tuft, goblet, Paneth, or enteroendocrine (EE) cells; and (2) Absorptive progenitors, which can differentiate into enterocytes or M cells (Figure 1). It is important that these signaling processes remain intact and efficient in order to maintain the proper ratio of absorptive to secretory cells, thus providing proper epithelial function. If these pathways are disturbed, inflammation can ensue and may contribute to a variety of gastrointestinal perturbations such as penetrating Crohn’s disease (Ortiz-Masià et al., 2020) and colorectal cancer (Meng et al., 2020).

Historically, two distinct types of ISCs have been distinguished by their position in the crypts and their expression of distinct markers: Leucine-rich repeat-containing G protein-coupled receptor 5 (Lgr5^+^) stem cells, which are fast cycling ISCs; and slow-cycling +4 stem cells (so named because of their position as the fourth cell from the crypt base), which are radioresistant and considered to have ISC potential. These cells express the markers homeodomain-only protein homeobox (Hopx) (Takeda et al., 2011), telomerase reverse transcriptase (Tert) (Breault et al., 2008; Montgomery et al., 2011), polycomb complex protein Bmi-1 (Bmi1) (Sangiorgi and Capecchi, 2008), and leucine-rich glioma inactivated 1 (Lrgi1) (Powell et al., 2012), which are robustly expressed by *Lgr5*^+^ CBCs (Muñoz et al., 2012) (Figure 1). It should be noted that +4 cells have reduced labeling efficiency of daughter cells during long-term lineage tracing studies, compared to Lgr5^+^ cells. Thus, the notion of +4 cells being deemed stem cells has been challenged recently. Moreover, the +4-position terminology restricts the nomenclature of regenerative cells and discounts the roles of regenerative stem cells in other crypt locations. Because of the rapidly evolving understanding of what these cells are, in this review we will refer to the +4 position regenerative cells using the designations by which they were used by the authors of the respective studies that refer to them: i.e., +4 cells, +4 stem cells, regenerative cells, damaged associated, cells, etc.

Recent literature has highlighted the potential roles of both Lgr5^+^ IECs, “+4 cells,” and other cells derived by de-differentiation of more mature cells as the DARSC population, as they all have been shown to regenerate during the healing process and provide protection. The following sections will highlight recent literature on these distinct cell types, their differences and similarities, and how they might function in the setting of chronic inflammation.

## Lgr5^+^ ISCs: The Infantry and the Reserve?

**FIGURE 2:**
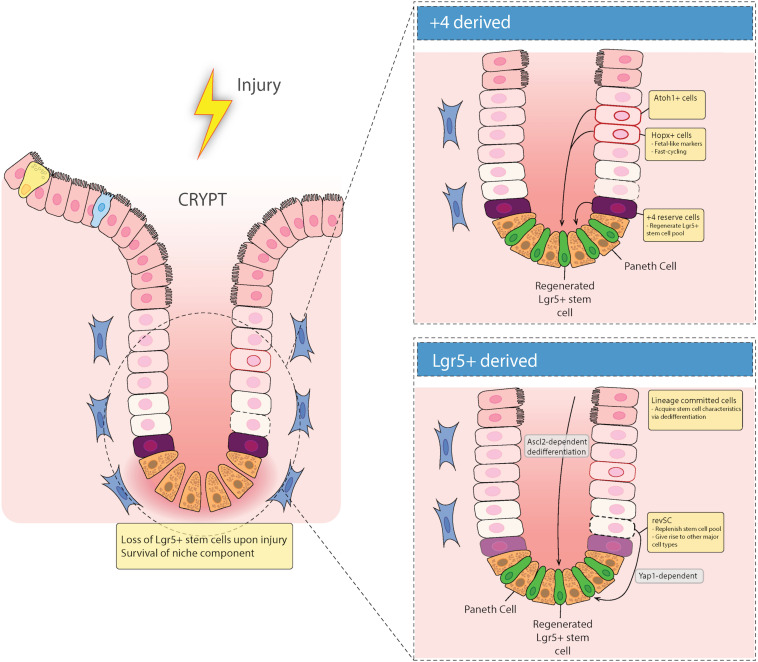
Epithelial repair response to injury. Recent evidence has suggested that there are two types of regenerative cells that fill in the damaged epithelium after injury. When the intestinal epithelium is damaged, either acutely (e.g., via irradiation), or chronically (e.g., in inflammatory bowel diseases), there can be loss of Lgr5^+^ stem cells in the crypt base. These gaps in the epithelium are filled in with either Lgr5^+^-derived reserve stem cells that can de-differentiate downward, or are +4 cell position-derived, including Hopx and/or Atoh1 expressing mature cell precursors that de-differentiate and migrate down from the TA zone to regenerate the stem cell niche.

While Lgr5^+^ crypt base columnar cells (CBCs) drive turnover and differentiation into mature epithelial cells (Barker et al., 2007) the intestinal epithelium is able to recover following injury to these CBCs (Metcalfe et al., 2014). This suggests that there are reserve stem cells (RSCs) that replenish abolished Lgr5^+^ stem cells. Cells in the +4 position, discussed in more detail below, have generally been considered the main RSCs (Montgomery et al., 2011; Takeda et al., 2011; Powell et al., 2012; Yan et al., 2012). However, recent literature suggests that Lgr5^+^ cells can emerge from de-differentiated epithelial cells during injury to provide epithelial restitution, thus raising the idea that +4 position cells do not migrate to the site of injury to rescue their damaged counterparts (Murata et al., 2020) but rather the damaged epithelium is replenished by newly emerged proliferative cells that express Lgr5 (Figure 2).

Recent evidence for the ability of Lgr5^+^ cells to act as DARSCs following radiation injury was shown by Ayyaz et al. (2019). They found that irradiation induces a distinct cell type marked by high expression of *Clu*, which encodes stress-response genes that are relevant in cell survival (Zhang et al., 2014). To understand the role of *Clu*^+^ stem cells during homeostatic turnover, they used a tamoxifen (TAM)-inducible label system to show that *Clu*^+^ cells give rise to both Lgr5^+^ CBCs and differentiated progeny cells. Moreover, they found that *Clu*^+^ single cell cluster 2 (SSC2) cells were able to reconstitute the damaged Lgr5^+^ ISC niche to revival crypt cell populations (revSCs), in both acute and chronic dextran sodium sulfate (DSS) colitis mouse models. Most importantly, they found that diphtheria toxin receptor (DTR)-mediated selective depletion of *Clu*^+^ cells resulted in failure of intestinal regeneration after DSS colitis, in a Yap1 dependent manner (Ayyaz et al., 2019) (Figure 2). These data refute previous reports that suggests DARSCs as being exclusively +4 position derived, and supports the idea that in a state of damage, reserve RSCs are derived from Lgr5^+^ cells. However, since 25% of these *Clu*^+^ revSCs are positioned in the +4 position, this distinction remains uncertain.

While this study proved that DARSCs can be derived from Lgr5^+^ cells, Murata and colleagues set out to understand if Lgr5^+^-derived progeny cells can de-differentiate to replenish the damaged Lgr5^+^ ISC niche (Murata et al., 2020) (Figure 2). *Ascl2*, a basic-helix-loop-helix transcription factor gene, is a transcriptional target of the Wnt signaling pathway, and is restricted to basal crypt cells in mice (Jubb et al., 2006). With this in mind, Murata and colleagues utilized a TAM-inducible CRE system to permanently GFP label Lgr5^+^ cells and their progeny, and also to selectively deplete *Ascl2*^+^ cells through expression of DTR. They also employed *Ascl2* KO organoids. Using these methods, they discovered that *Ascl2* expression is essential for crypt cell de-differentiation after ISC injury (Murata et al., 2020). Moreover, after complete depletion of Lgr5^+^ ISCs using γ-irradiation, they demonstrated that DARSCs originate from Lgr5^+^ (GFP^+^) progeny, via de-differentiation. This implies that there is no recruitment from cells in the +4 position to the damaged epithelium. These two studies together indicate that Lgr5^+^ cells can act as DARSCs; although they conflict in terms of whether this is primarily due to preservation of a small population of cells, or de-differentiation of their progeny. Additional studies may be required to resolve this discrepancy.

While there is controversy regarding the origins of DARSCs, even less is known about inflammatory signals that direct them. Previously, IL-11 derived from myofibroblasts was shown to be necessary for regeneration in the intestinal mucosa (Bamba et al., 2003). However, this cytokine has never been studied in the context of which specific cells in the crypt respond to IL-11. Murata et al. (2020) showed that healthy ISCs express little *Il11ra1*, whereas *Ascl2-* expressing regenerative cells have increased levels of *Il11ra1.* IL-11ra1, an IL-11 receptor, and recombinant IL-11, both Ascl2 target genes, enhance crypt regeneration potential. A more recent study found that type I interferons impair mouse recovery from DSS colitis and the ability to form enteroids *in vitro* (Minamide et al., 2020). Epithelial-specific deletion of interferon-regulatory factor 2 (Irf2), which downregulates type I IFN signaling, led to loss of Lgr5^+^ ISCs and increased proliferation, suggesting a mechanism for this susceptibility, and also indicating another potential effect of inflammatory injury on the gut. Additional studies will be needed to understand the contribution of IL-11, interferons, and other inflammatory signals to DARSC development and maintenance.

## The Role of +4 Position Cells and Secretory Precursors in Repairing the Damaged Intestinal Epithelium

Situated just above the last Paneth cell in the crypt are the +4 position cells, which have a unique transcriptional profile including expression of Hopx (Takeda et al., 2011), Tert (Breault et al., 2008; Montgomery et al., 2011), Bmi1 (Sangiorgi and Capecchi, 2008), and Lrig1 (Powell et al., 2012) (Figure 2). These cells have previously been deemed the RSC population, which reconstitutes Lgr5^+^ CBCs during a state of injury (Breault et al., 2008; Sangiorgi and Capecchi, 2008; Montgomery et al., 2011; Powell et al., 2012), and have been previously shown to be radiation resistant (Tao et al., 2017; Montenegro-Miranda et al., 2020; Sheng et al., 2020), suggesting that these cells can survive acute injury in the crypt and fill in for their damaged counterparts. However, recent literature has underscored the complexity of characterizing these cells and called into question their “stemness.” This section will discuss the current literature on the different subsets of LGR5^–^ reserve stem cells (including “+4 cells”) and their ability to restore intestinal epithelial homeostasis post DNA damage and injury.

### Importance of Hopx and Atoh in Regenerative Cell Function and ISC Renewal

The homeodomain-only protein homeobox (Hopx) is a non-DNA-binding homeobox protein expressed in various tissue stem cell populations, including in the intestinal crypts. Takeda et al. demonstrated that the majority of the so-called label retaining cells in the intestinal crypt following irradiation injury (those retaining BrdU, indicating ongoing proliferative capacity) reside in the +4 position and express Hopx (Takeda et al., 2011). Moreover, they exhibited a bi-directional lineage relationship: Hopx*-*expressing cells can propagate to generate Lgr5^+^ ISCs and all mature epithelial lineages, while Lgr5^+^ cells can give rise to +4 position Hopx^+^ cells. This suggests they have an important role as RSCs. However, other recent studies have identified a distinct colitis-associated regenerative stem cell (CARSC) population that expresses Hopx in mouse models of colitis (Wang et al., 2019). Utilizing monolayer cultures in an air-liquid interface (ALI), which leads to maturation of the monolayers, epithelial injury was induced by submerging the monolayer in media mimicking a hypoxic and ER-stress mediated damaging environment. These damaged monolayers contained fast cycling Hopx^+^ regenerative stem cells characterized by the expression of fetal-like marker Tacstd2 (Trop2), thus distinguishing them from both Lgr5^+^ cells and slow-cycling Hopx^+^ +4 position cells (Wang et al., 2019). It is quite possible that these Hopx^+^ expressing cells could be derived from other secretory derived lineages, because Hopx^+^ regenerative stem cells co-express goblet and EE cell signatures. However, most notably, they also express the secretory IEC lineage marker Atonal homolog-1 (Atoh, also known as Math1 in mice), which is the master transcription factor for secretory IECs (Yang et al., 2001). Atoh has recently been shown to be necessary for plasticity of secretory progenitors and tissue regeneration (Tomic et al., 2018), and these cells have the ability to repair the epithelium during DSS-induced colitis (Ishibashi et al., 2018; Tomic et al., 2018; Castillo-Azofeifa et al., 2019). Thus, Atoh^+^ secretory progenitor cells may contribute to the development of Hopx^+^ regenerative cells during colitis (Figure 2).

Atoh1^+^ cells are essential for secretory cell differentiation, as Atoh1 deletion in mice leads to development of intestines with a grossly normal crypt-villus architecture, but that are entirely populated by enterocytes (van Es et al., 2010). Atoh1 expression in populations of +4 position cells marks them as secretory lineage precursors, but recent evidence suggests that these Atoh1^+^ progenitors have the capacity to self-renew and give rise to multiple lineages with high frequency during homeostasis, and are able to contribute to colonic regeneration after γ-irradiation or DSS-induced injury (Buczacki et al., 2013; Ishibashi et al., 2018; Tomic et al., 2018; Yu et al., 2018), thus highlighting their importance in the differentiation process during the repair stage after intestinal damage, in the large intestine. However, Atoh is dispensable for regeneration in the SI (Durand et al., 2012), suggesting that the mechanism of regeneration involving Atoh1 may be tissue specific. The exact mechanisms of epithelial regeneration were studied by Tomic et al. (2018), who found that multi-site phosphorylation of Atoh1 results in the inhibited ability to self-renew, thus leading to blunted ability to regenerate proper clones during damage.

To understand whether Atoh1^+^ cells are dependent on *Lgr5*^+^ stem cells for epithelial repair, Castillo-Azofeifa et al. (2019) performed genetic fate mapping using *Lgr5*^GFP–IRES–CreERT2^; *ROSA26*^tdTomato^ mice undergoing DSS-induced colitis. They discovered that after DSS-induced injury, Lgr5^–^ Atoh1*^+^* secretory progenitors, not Notch1^+^ absorptive progenitors, provided epithelial repair. These data suggest that Atoh1^+^ cells are essential in maintaining ISC function after injury and can differentiate into distinct mature cells types that protect the epithelium after acute damage.

*Bmi1*^hi^ and *Tert*^+^ populations, which contain mostly EE cells (Yan et al., 2012, p. 1), and rare “reserve” ISCs, as well as both secretory and enterocyte progenitors (Tetteh et al., 2016; van Es et al., 2019), and even Paneth cells (Schmitt et al., 2018; Yu et al., 2018; Jones et al., 2019), have been shown to aid in Lgr5^+^ recovery of the epithelium. With this in mind, these studies did not look at the regenerative efficiencies of secretory and EE cells, and whether or not their ability to restore the damage epithelium is distinct in terms of (1) The speed of transit to the site of inflammation, and (2) their overall restorative capacity. This would be an interesting observation, as Yan et al. (2017) demonstrated that *Prox1*^+^ EE lineage cells during homeostasis can propagate into reserve injury-inducible ISCs.

Harnack et al. (2019) studied the role of the Lgr5 ligand R-spondin 3 (Rspo3), which potentiates Wnt signaling, in establishing the ISC niche during homeostasis or colonic injury. Lacking Paneth cells, colonic crypts rely on mesenchymal cell production of Wnt-supporting ligands like R-spondin. In this study, myofibroblast-derived Rspo3 was found to be necessary and sufficient for maintaining Lgr5^+^ cells during homeostasis. However, in the context of injury, myofibroblasts do not maintain Lgr5^+^ cells, and instead interact with Lgr4 expressing cells, to generate new crypts. Mice lacking Rspo3 have a blunted ability to repair the damaged epithelium. It is important to note that these Axis inhibition protein 2 (Axin2^+^) cells express high amounts of *Atoh1*, thus raising the possibility that Axin2^+^ Lgr5^–^ regenerative stem cells are derived from secretory precursors. Moreover, deletion of Rspo3 completely prevented crypt regeneration during DSS colitis. Interestingly, this was shown not to require Lgr5^+^ cells or “reserve” stem cells (in this case, marked via expression of Axin2 and Atoh), both of which were depleted by DSS. Instead, Rspo3 was needed to support a population of differentiated Krt20^+^ enterocytes that expressed the alternative Rspo receptor Lgr4, allowing them to de-differentiate (Harnack et al., 2019). This study provides additional evidence that differentiated IECs can repopulate a damaged ISC niche, and possibly offer intestinal restorative properties.

These data collectively suggest that Atoh1^+^ cells have the characteristics of (1) Multipotency in self renewal capacity and (2) The ability to promote colonic repair and epithelial regeneration during colitis. Hence, they could be targeted for future therapies to resolve colonic injury. Having the ability to resect tissues from patients with acute or chronic inflammatory intestinal diseases, grow ISCs in a dish as enteroids, and genetically modify them to express *Atoh1*, or to sort Atoh1^+^ cells to proliferate as enteroids, could allow for clinicians to engraft these ISCs back into the damaged areas of the epithelium to promote expansion of cells that will aid in a more robust colonic repair to resolve chronic injury. One limitation to this hypothesis is that overexpression of Atoh1 has been shown to increase secretory cell differentiation, thus reducing their regenerative potential. However, using a Tryptophan hydroxlayse 1 (*Tph1*)-CreERT2 mouse model, Sei et al. (2018) recently showed that EECs, specifically enterochromaffin cells (EC), have demonstrated stem cell potential and have the ability to dedifferentiate and act as a RSC during irradiation. Hence, Atoh1-mediated secretory differentiation does not preclude subsequent de-differentiation to repair ISC injury.

These studies also support the hypothesis that Atoh expressing cells can be targeted for colonic inflammatory diseases. Hopx expressing cells can possibly be used in a setting of chronic inflammatory diseases mediated by hypoxia or endoplasmic reticulum (ER) stress, such as IBD (Giatromanolaki et al., 2003; Kaser et al., 2008; Shah et al., 2008; Vanhove et al., 2018; Rees et al., 2020b), as the authors were able to show that these cells rapidly proliferate to provide protection in response to hypoxic injury, which upregulates the ER stress cellular protection pathway known as the unfolded protein response (UPR). The UPR has been shown to be important in maintaining ISC proliferation (Heijmans et al., 2013), and can be an important pathway to target for the repair of IECs in chronic intestinal diseases.

### Msi1 and TIGAR: Boosting the Potential of DARSCs

One concern with “+4 cells” and possibly other DARSCs is that their slow cycling in a homeostatic state means that they need to receive signals to increase their proliferative potential at a rate fast enough to preserve the intestinal barrier following severe injury, such as following lethal irradiation. Two recent factors that may contribute to this are Musashi homolog 1 (Msi1) and TP53-induced glycolysis and apoptosis regulator (TIGAR).

Msi1 has previously been identified as a marker of ISCs (CBCs, and +4 position cells) (Kayahara et al., 2003; Li et al., 2015). Sheng et al. (2020) set out to understand if Msi1 can rapidly cycle to restore a damaged intestinal epithelium. This was investigated by generating an Msi1CreERT2 allele for lineage tracing analysis and this led to the discovery that Msi1^+^ cells are indeed positioned in the +4 position in the intestinal crypt and are resistant to DNA damage via y-irradiation. Using ssRNA-seq, they demonstrate that Msi1^+^ cells have low-to-negative Lgr5 expression, and repopulate the damaged intestinal epithelium before Lgr5^high^ emergence, suggesting that Msi1^+^ cells are more rapidly cycling than Lgr5^high^ cells. This stands in contrast to the current hypothesis that +4 position cells function as RSCs that restore depleted Lgr5^high^ CBCs first, thus allowing CBCs to rapidly divide to repair the damaged intestinal epithelium. Interestingly, Msi1+ cells move both upwards and downwards along the crypt villus axis, post TAM induction, which suggests that Msi1^+^ cells can differentiate into CBCs, Paneth cells, and villus defining cells. These data suggest, and further support earlier work, that cells of the secretory lineage are capable of restoring a damaged intestinal epithelium. This work supports previous work by Carroll and colleagues that demonstrates while most Lgr5^+^ cells are thought to be continually proliferative, residing in a licensed state, ∼20% of Lgr5^+^ cells remain in an unlicensed G_1_ cell cycle phase (Carroll et al., 2018).

One possible signal for Msi1^+^ to initiate repair is arachidonic acid (AA). Previous work showed that AA may contribute to proliferation of ISCs (Hiraide et al., 2016). More recently, Wang and colleagues investigated the role of AA on intestinal regeneration post y-irradiation. They discovered that AA promotes the proliferation of SI epithelial cells post irradiation in an *Ascl2I* and WNT signaling manner. Most notably, AA’s regenerative effect was mediated via the regulation of Msi1+ radiation resistant cells, not Lgr5^+^ cells, but the exact mechanism is not known (Wang et al., 2020).

TIGAR is a protein induced in mouse intestinal crypts by c-Myc. Under homeostatic conditions, suppression of the β-catenin/c-Myc axis within +4 position slow cycling ISCs leads to limited regenerative responses to restore intestinal integrity after injury. Chen et al. recently showed that restricted overexpression of TIGAR in Bmi1^+^ cells, but not Lgr5^+^ cells, was able to reverse lethal irradiation injury in mouse SI, by activating AP-1 signaling to induce proliferation (Chen F. et al., 2020).

Together, these data suggest that +4 position cells can play an important role in the regeneration of damaged small IECs, where they can provide several advantages compared to Lgr5^+^ cells that de-differentiate to rescue the damaged epithelium. (1) Msi1^+^ cells positioned in the +4 position cycle faster than Lgr5^+^ cells during DNA damage, migrating both downward toward the crypt and apically toward the villus region; (2) Msi1^+^ cells facilitate rapid tissue repair via regulation by AA, a fatty acid released by phospholipase A2 and the critical precursor for prostaglandins, both heavily involved in IEC inflammatory signaling; and (3) Induction of TIGAR in Lgr5^–^ DARSCs can reverse their slow proliferative phenotype, facilitating rapid division and restoration of the intestinal barrier. Future studies in human cells or enteroids will be needed to determine if these observations can be generalized to human diseases.

### Hypoxia and ER Stress as Drivers of Epithelial Repair and Differentiation

The process of cellular differentiation and proliferation in the intestinal crypt involves an abundance of secretory protein processing in the ER, which puts a considerable metabolic burden on the cells. These proteins can be improperly processed, misfolded, or inadequately glycosylated, thus leading to ER stress. The UPR, which is activated during ER stress to restore the cell to homeostasis, is also essential in determining stem cell fate and differentiation (Heijmans et al., 2013). The mechanisms of how hypoxic injury and ER stress affect ISC function and intestinal repair following injury are an area of active investigation.

Hepatocyte nuclear factor 4 alpha (HNF4α) is expressed in intestinal villi (Stegmann et al., 2006) and has been identified as a transcription factor that regulates the expression of genes during intestinal cellular differentiation during endoderm development (Yao et al., 2016). It was recently discovered that HNF4α is involved in the activation of ER stress during intestinal epithelial differentiation (Tunçer et al., 2019), demonstrated by the upregulation of XBP1 and ATF6 (two ER stress mediators) downstream of HNFα activity. This study is important because it ties together the importance of ER stress in maintaining proper ISC proliferation and differentiation (Heijmans et al., 2013) with the fact that HNF4α is expressed in the intestinal villi (Stegmann et al., 2006), which suggests that these two signaling pathways are essential for ISC fate and fitness.

In support of this, our group recently published that ER stress in colon-derived enteroid monolayers drives inappropriate TLR5 responses leading to the expression of unidentified factors that mature dendritic cells (DCs) to become pro-inflammatory—describing a novel pathway that may lead to the inappropriate anti-commensal inflammatory responses seen in IBD (Rees et al., 2020b). Arguably the most important finding of this study was that IBD derived enteroids have blunted cytokine responses to the TLR5 agonist flagellin (FliC), and this was found to be driven by dysregulated ER stress pathways in the IBD enteroids, but not TLR5 expression levels, compared to healthy controls. This raises the hypothesis that prolonged periods of ER stress can alter the ISC niche, permanently changing their phenotype and perpetuating inflammation and disease.

Most recently, Montenegro-Miranda et al. (2020) established an *in vitro* enteroid damage-repair model that identified HNF4α is an essential regulator of intestinal epithelial repair following γ-irradiation damage. They produced VillinCre X Hnf4^fl/fl^ or Hnf4-KO mice enteroids, and assessed their ability to grow and proliferate. They found that HNF4α KO enteroids were unable to propagate and grow *in vitro* compared to their littermate control-derived enteroids. Most notably, they found that HNF4α is important for epithelial regeneration *in vivo* and HNF4α-KO mice have an increase in the secretory differentiation lineage. The blunted ability to regenerate the epithelium was linked to the inability to keep up with the metabolic demands of regenerating the ISC niche after γ-irradiation (Montenegro-Miranda et al., 2020). This could be due to effects on ISC metabolism, as Hnf4α and the related protein Hnf4γ were recently shown to be required for fatty acid oxidation in mouse ISCs, and conditional double knock-outs lost the ability to renew the ISC compartment (Chen L. et al., 2020).

These studies have particular significance in the field of IBD since it was discovered that *HNF4α* is a risk allele in ulcerative colitis, with the disease allele associated with reduced function (UK IBD Genetics Consortium et al., 2009; Marcil et al., 2012). With this in mind, it is appropriate to speculate that in a chronic setting of inflammation, as seen in UC, HNF4α is essential in maintaining barrier integrity during homeostasis and acute injury, although it is not essential for the expression or maintenance of Lgr5^+^ cells (Montenegro-Miranda et al., 2020).

Studying the ISC niche during homeostasis and disease (acute or chronic) will be an important topic for future researchers to better understand how inflammation and dysregulated signaling pathways may led to the development of DARSCs, and whether or not these cells are beneficial in the chronic setting. Although it has been shown in an acute ER stress-driven setting that Hopx expressing RSCs can provide protection, it remains to be seen if these cells, whether derived from the +4 position or not, can provide protection in a chronic setting. One would postulate that chronic inflammation would lead to the prolonged development of either Atoh1^+^, Hopx^+^, or Hnf4α^+^ cells in the crypt, thus providing short term epithelial maintenance. However, how long can these cells or their progeny remain in the crypt? Does this ultimately not allow for the expansion of healthy Lgr5^+^ cells once inflammation has resolved? These questions need to be addressed in order to better understand the nature of a true homeostatic ISC niche post inflammatory disease.

In summary, these studies suggest that there are several redundant layers of protection against permanent injury following acute intestinal crypt injury. Lgr5^+^ cells, “+4 cells” and Lgr5^+^ progeny that de-differentiate from secretory or absorptive lineages can all help maintain epithelial barrier integrity during a state of acute inflammation. Future studies should focus on which specific secretory or absorptive cells are responsible/most efficient in replenishing the damaged epithelium in acute models, as well as in a chronic setting. One important question that remains to be addressed is whether RSCs are just as efficient in providing barrier integrity, proper paracrine signaling, and appropriate innate and adaptive immune cross-talk during infection and other inflammatory states, compared to their parental counterparts. This information can lead to future therapeutics to alter cell signaling pathways that allow for the development of distinctive cell populations in order to restore barrier integrity and intestinal epithelial homeostasis. One key to these therapeutics will be to better understand regulation of IEC differentiation and plasticity. The following section will cover the genetics behind differentiation in ISCs to highlight some future directions in the field of IEC plasticity.

## How Differentiated Is Too Much Differentiation—Is There Any Going Back?

As mentioned previously, there is emerging evidence that transit amplifying (TA) cells and even fully differentiated IECs may be able to de-differentiate and replenish the damaged ISC niche. There are important questions that need to be addressed about these cells: (1) Are precursors of differentiated cells (goblet cells, Paneth cells, EE, and absorptive enterocytes), innately destined (based on their location) to differentiate into their mature forms?; and (2) How do epigenetic changes result from the environmental signals that drive their maturation? Key signaling molecules, *Wnt*, *Bmp*, and *Notch*, which are produced by Paneth cells and myofibroblasts (Kosinski et al., 2007; Hughes et al., 2011; Sato et al., 2011) regulate ISC fate and function (Rees et al., 2020a). Wnt expression has been shown to be regulated in part by histone variant functions, so it is of importance to study possible epigenetic factors, as well as chromatin remodeling factors that determine the fate of the Lgr5^+^ ISC niche. The following sections will discuss emerging literature on permissive chromatin and IEC transcription factors that may underlie IEC plasticity and repair following injury.

### Chromatin Structure and IEC Plasticity

Chromatin structural modifications lead to repression or activation of certain genes that are necessary for self-renewal and differentiation (Hager et al., 2009; Skene and Henikoff, 2013). An open chromatin structure may also facilitate epigenetic modifications allowing for de-differentiation. Kim et al. (2014) demonstrated that mouse small intestinal ISCs have unusually open or “permissive” chromatin structures that allow lineage-defining transcription factors (e.g., Atoh1) to lead to differentiation, while removal of these transcription factors in the setting of permissive chromatin can reverse this; this may underlie the plasticity of IECs in response to injury. This notion was supported more recently by Jadhav et al. (2017), who used *Bmi1*^GFP^ mice to demonstrate that preterminal EE cells, as well as a goblet cell precursor population defined by the markers CD69^+^CD274^+^, have the ability to de-differentiate into Lgr5^+^ cells upon ISC ablation via their dynamic chromatin accessibility, which remains open during injury.

One particular histone variant, H2A.Z, has been shown to remodel chromatin structures, leading to gene expression changes. The function of H2A.Z in ISC differentiation and renewal has recently been reported. SNF-2 related CBP activator protein complex (SRCAP) components YL1 and Znhit1 have been shown to regulate the incorporation of H2A.Z into the chromosome (Cai et al., 2005; Latrick et al., 2016; Liang et al., 2016). However, the exact mechanisms behind the roles of Znhit1 and YL1 in the SRCAP complex and how they modulate H2A.Z function was unknown. To address these questions, Zhao et al. (2019) established a *Znhit1* KO mouse model to study its role in intestinal epithelial establishment and maintenance. They found that Znhit1 regulates the expression of *Lgr5*, *Tgfb1*, and *Tgb12*, via the incorporation of H2A.Z into the transcriptional start site (TSS) regions of the aforementioned genes. Znhit1 deficiency leads to a downregulation of Lgr5 and an activation of TGFβ signaling, which mediates self-renewal capacity and drives differentiation of Lgr5^+^ ISCs. It is important to note that Znhit1 deletion altered only *Lgr5* expression without affecting Wnt signaling, as there were no genetic expression changes in *Ascl2*, a Wnt-targeted master transcription factor for activating the transcription of *Lgr5* (van der Flier et al., 2009), or *Axin2*, which is an activity indicator of Wnt signaling (Lustig et al., 2002). These data suggest that Znhit1 is essential for the differentiation outcomes of Lgr5^+^ ISCs, and raises the question that targeting Znhit1 or the SRCAP as a whole could restore intestinal homeostasis in diseases that perturb the ISC niche. This thought could be further supported by a recent study that demonstrates that SCRAP promotes self-renewal of mouse ISCs (Ye et al., 2020).

Several additional chromatin-modifying proteins and transcription factors have recently been identified as helping to control ISC fate and IEC differentiation. One recent study showed that the transcription factor Id3 is likely responsible for maintaining ISC fate via repression of open chromatin regions (Raab et al., 2020). Transcriptional co-repressors MTG8 and MTG16, were recently been shown to be involved in regulating ISC fate into secretory lineages by indirect Notch signaling suppression, via ATOH (Baulies et al., 2020). These two chromatin modulators are expressed by early progenitor cells that are located in the +4/5 position along the crypt villus axis. Polycomb-repressive complexes (PRCs) repress transcription via compacting of chromatin and limiting DNA accessibility. There are two major types of PRCs, PRC1 and PRC2. PRC 1 was shown to be crucial for maintaining stem cell identity via the preservation of Wnt/β-catenin activity (Chiacchiera et al., 2016a). PRC1 is essential for maintaining stem cell self-renewal, but in contrast, PRC2 was shown to be dispensable for intestinal regeneration (Chiacchiera et al., 2016b), yet is sufficient for maintaining cellular plasticity and epithelial regeneration in the crypt bottom after radiation-induced damage.

In conclusion, ISC plasticity is tightly regulated via multiple tissue-specific transcription factors and chromatin modifiers. Studies of chromatin accessibility in ISCs during various forms of intestinal injury, including chronic diseases like IBD, could lead to important interventions to help maintain normal ISC function following chronic injury.

### Control of IEC Fate After Injury by CDX2 and the Hippo Pathway

The process of migration of stem cell progeny cells from the crypt into the villus, thus leading to their differentiation into specific secretory or absorptive lineages, is controlled by transcription factors, such as members of the CDX homeobox gene family (Xu et al., 1999; Beck, 2004), namely, CDX1 and CDX2, which are essential for intestinal maintenance and compartment-specific differentiation (Hryniuk et al., 2012). p400 ATPase participates in the incorporation of H2A.Z (Kobor et al., 2004; Gévry et al., 2007), and Rispal et al. set out to understand if there was a link between p400 induction of Wnt (via CDX2) and H2A.Z dynamics. They discovered that H2A.Z modulates the expression of intestinal progenitor differentiation by preventing terminal differentiation, thus acting as a negative regulator of intestinal differentiation (Rispal et al., 2019). This was demonstrated using a *H2A.Z* KO mouse model, which led to the inhibition of mRNAs of differentiation markers of enterocytes (*sucrase-isomaltase*), and goblet cells (*Muc2* and *Muc4*). Moreover, the ability of CDX2 to repress transcription, via binding to target gene promoters, was shown to be blunted by H2A.Z incorporation into the chromatin. Taken together, these data show that H2A.Z may be an important link between Wnt signaling and IEC differentiation, acting through CDX2.

One hypothetical role of CDX2 in signaling and IEC differentiation could be a direct interaction with HNF4α. CDX2 has recently been shown to interact with transcription factors such as GATA4 and HNF1α, which regulate the expression of differentiation markers sucrase-isomaltase, lactase-phlorizin hydrolase, and μ-protocadherin (Boudreau et al., 2002; van Wering et al., 2004; Hinkel et al., 2012). A recent study using chromatin immunoprecipitation (ChIP) showed that half of DNA binding sites of HNF1 are shared with HNF4α also, suggesting that similar gene regulation may occur for both transcription factors (Yang et al., 2016). This may be particularly relevant given that HNF4α has a role in determining the fate of IECs, mainly, enterocyte differentiation and identity (Chen et al., 2019), and evokes the UPR during ER stress, which is an essential pathway in maintaining proliferation of the ISCs and differentiation of their progeny (Heijmans et al., 2013). Moreover, CDX2 has been shown to regulate the expression of Wnt inhibitors *APC* and *AXIN2* (Olsen et al., 2013). Studying CDX homeobox gene family members may therefore be essential in targeting transcription factors to induce the differentiation of specific ISCs to provide barrier maintenance.

The studies discussed above highlight the possibility that if one targets the transcriptional machinery of secretory or absorptive derived IECs, no matter how differentiated they may be, they may be able to de-differentiate and migrate toward the crypt to provide epithelial maintenance. Future research should study the efficiency of targeting the transcriptional machinery of the aforementioned cells to help repair injury *in vivo*, or potentially to generate robust, therapeutically efficient clones of cells for autologous organoid transplantation.

Another important set of proteins essential in regulating cell-specific proliferation, survival and fate determination in the gut is known as the Hippo pathway. It was first described in *Drosophila* as being required for controlling organ growth, and is implicated in cancer development. Most notably for this review, the mammalian Hippo pathway is also involved in maintenance of intestinal structure and prevention of carcinogenesis, and more recent studies have implicated it in injury repair.

The Hippo pathway in mammalian cells is composed of MST and LATS kinases, which are responsible for inactivating two cellular proliferation, survival and fate transcriptional regulators, YAP and TAZ. Yap in mice has previously been shown to be involved in regenerative cell signaling via repression of Wnt target and ISC genes (Axin 2, Lgr5, Olfm4) as well as Paneth cell differentiation suppression (Lyz, Kit, Wnt3, Math1) (Barry et al., 2013; Gregorieff et al., 2015). Notch signaling drives the proliferation and differentiation of secretory cells into Paneth cells (VanDussen et al., 2012) and it has recently been shown that there is cross-talk between Hippo and Notch signaling pathways. Serra et al. (2019) recently showed that Yap1 activation initiates Notch/DLL1 lateral inhibition, thus driving Paneth cell differentiation and crypt formation from nascent mouse enteroids. More recently, Li et al. (2020) produced an IEC-restricted Lats1/2 knockout mouse, and showed that it led to loss of ISCs coupled with Wnt-uncoupled crypt expansion, in a mechanism dependent on Yap/Taz. These data collectively suggest that Hippo signaling is essential for fate determination of ISC progeny and may be a significant target for regenerative stem cell therapy, yet these exact mechanisms need to be further investigated.

It was previously demonstrated that Yap and Taz are dispensable for Wnt signaling under homeostatic conditions (Azzolin et al., 2014; Gregorieff et al., 2015), suggesting that the Hippo pathway is not a major signaling pathway involved in cellular plasticity, but it is nonetheless important in determination of cellular fate. Hippo and Yap cross-talk pathways have been shown to converge with Wnt, BMP, TGF-β, Notch, and EGF (reviewed in Hansen et al., 2015). As we know, BMPs promote the differentiation of ISCs toward mature IEC phenotypes, and mature differentiated epithelial cells can also produce BMPs (Haramis et al., 2004; Batts et al., 2006; Qi et al., 2017). Wnt ligands are mainly expressed by Paneth cells and mesenchymal cells surrounding the crypts (Farin et al., 2012; Kabiri et al., 2014) and recently IEC monolayers were shown to be able to control their own growth and organization through a WNT and BMP feedback loop (Thorne et al., 2018).

These data prompted the study of the Hippo pathway’s involvement in suppressing Wnt activity via BMP/TGF-β signaling. Bae et al. (2018) showed that IEC-specific deletion of MOB1A/B, an important kinase involved in activation of Lats kinase and subsequent phosphorylation of Yap, led to lethal intestinal degeneration and loss of ISCs via BMP and TGFβ-induced suppression of Wnt activity, showing a requirement for Yap in mouse intestinal homeostasis.

Collectively, these studies suggest the importance of the Hippo pathway in influencing IEC homeostasis by Wnt suppression, ultimately leading to cellular plasticity modulation. Relevant for this review, there has also been recent work examining the roles of LATS and YAP/TAZ in recovery from intestinal injury. The first such study was by Gregorieff et al. (2015), who found that recovery from radiation injury in mice required Yap activity to suppress Wnt signaling and activate Egf (epidermal growth factor) pathways to induce ISC survival and division. At the same time, this Yap/Egf signaling was also required for tumor development in Apc^–/–^ mice; this suggests a molecular mechanism whereby the repeated cycles of injury and repair in IBD can promote carcinogenesis. A similar proliferative effect of Yap when Apc is deleted was shown in mouse enteroids by Azzolin et al. (2014). More recently, it was shown that Lats2 and Yap1 positively regulate each other’s expression during *H. pylori* infection, and that Lats2 activity restricts intestinal metaplasia, again supporting the idea that Yap1 may be involved in inflammation- or injury-induced carcinogenesis (Molina-Castro et al., 2019).

Four other recent studies have examined the role of the Hippo pathway on recovery from chemical-induced intestinal injury in mice. Yui et al. (2018) found that during recovery from DSS colitis, the colonic epithelium goes through a “primitive” or fetal-like state on its way to recovery, and that this transition requires Yap/Taz activity. Romera-Hernández et al. (2020) induced intestinal injury using methotrexate, and found that group 3 innate lymphoid cells (ILC3s) drive intestinal epithelial repair in a Yap1 dependent manner, but independent of intestinal regenerative IL-22. Xu et al. (2020) examined the role of the stromal cell protein ISLR [immunoglobulin (Ig)-like domain and five leucine-rich repeat (LRR) domains], in recovery from DSS and TNBS injury in mice. Islr was previously shown to maintain an undifferentiated state of stromal cells (Maeda et al., 2016). In this study, Xu et al. (2020) found that Islr inhibits Lats, leading to increased Yap activity. They then used a stromal cell conditional KO mouse model targeting Islr, in order to understand its role in modulating the Hippo signaling pathway during homeostasis and damage. They found that Islr is upregulated following DSS injury, and that stromal cell-specific deletion of Islr did not lead to a phenotype in uninjured mice, but impaired recovery following DSS or TNBS injury. Importantly, they also showed that ISLR is overexpressed in human tissues from patients with IBD and colorectal cancers, suggesting that inflammation drives the expression of ISLR. Finally, Lukonin et al. (2020) used a single cell imaging platform to identify a novel role of the orphan retinoid X receptor RXR in IEC proliferation and differentiation. They found that RXR activation using all-*trans* retinoic acid or a chemical activator led to maturation and differentiation of enteroids, associated with Yap cytosolic localization. In contrast, treatment with an RXR inhibitor let to reduced CDX activity and Yap nuclear localization. Moreover, RXR inhibition improved recovery from DSS colitis in mice. This study opens the door to pharmacologic treatments that can modify the Yap-mediated commitment to proliferation or repair, potentially blocking cycles of injury-mediated chronic damage.

Together, these recent studies underscore the importance of LATS/YAP/TAZ signaling not only in maintenance of intestinal homeostasis, but in repair from acute injury. Additional studies will be required to further assess how these pathways are affected in chronic states of inflammation such as in IBD. This could lead to approached to target mediators of the Hippo pathway in order to enhance restoration and repair of the intestinal epithelium, in such a way as to avoid overactivation of YAP and subsequent carcinogenesis. They could also open the door to new ways to generate reparative epithelia safely, which we discuss in the following section.

## Autologous Organoid Transplantation—Can We Rescue the Damaged Intestinal Epithelium?

**FIGURE 3:**
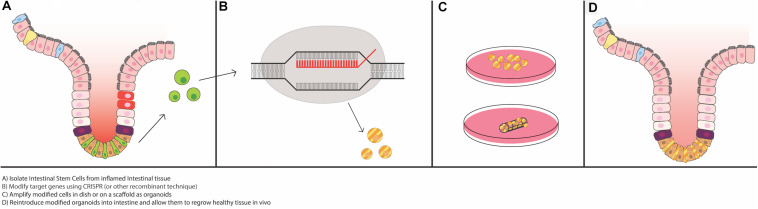
Potential for use of intestinal organoids as “grafts” for the damaged epithelium. Recent advances in epithelial regeneration via organoid transplantation have shown that it could be possible to recover a damaged intestine or colon with autologous organoid transplantation. One approach to improve efficacy in engraftment and recovery post damage would be to isolate intestinal stem cells (ISCs) from the inflamed tissue, or if possible, non-inflamed adjacent tissue, modify target genes via CRISPR or equivalent recombinant techniques, expand the modified ISCs *in vitro*, and engraft back into the damaged area to recover the injured area. Tissue scaffolds, either synthetic or derived from human intestinal resections, could facilitate growth and proper cellular alignment.

Despite recent advances in IBD treatment, the diseases remain incurable, and many patients fail to achieve remission even with biologic anti-cytokine therapies. Recent work suggests that there may be permanent changes in ISC function in IBD that underlie its chronic, relapsing nature. Specifically, it has been recently been shown via RNA sequencing and enteroid model experiments that there are distinct differences in gene expression profiles, inflammatory cytokine milieus, and epithelial cell phenotypes in healthy tissue compared to tissue from people with IBD (Obita et al., 2003; Mitsuhashi et al., 2005; Ito et al., 2013; Dotti et al., 2017; Ragland and Criss, 2017; Suzuki et al., 2018; Martin et al., 2019; Smillie et al., 2019; Rees et al., 2020b). With this in mind, it is imperative that researchers look for new methods/strategies to normalize and repair ISC function in IBD. After the rapid growth in technology to grow and maintain intestinal epithelial derived Lgr5^+^ stem cells as enteroids, studies of autologous organoid transplantation to re-populate the ISC niche have just started to take place. This type of methodology has been demonstrated using cancer models and has been extensively reviewed for use in the SI (Qi et al., 2020), and the possibility of it being used in IBD (Okamoto et al., 2020), although the major focus so far has been with use of organoids derived from induced pluripotent stem cells (iPSCs) and mesenchymal stem cells (MSCs). We will highlight the current literature on ISC enteroid transplantation in the context of replacing or aiding in the development of damage associated ISCs, and Figure 3 will illustrate methodologies to allow for successful enteroid transplantation into damaged intestinal epithelia (Figure 3).

To date, all of the studies that have performed ISC transplantation have used mouse models that allow for engraftment without rejection because of MHC histocompatibility issues. However, recently a model was published using xenographic human colon stem cell transplantation into NOD.cg-*Prkdc^scid^Il2rg^tm1Sug^*/Jic (NOG) mouse crypts (Sugimoto et al., 2018). Sugimoto et al. (2018) performed lineage tracing using CRISPR-Cas9 to engineer an Lgr5-CreER knock-in allele for engraftment into the NOG mice. To induce epithelial injury that allows for successful engraftment of the donor cells, they treated the recipient colons with EDTA, followed by scraping one side of the colonic epithelium, prior to engraftment with GFP-labeled human colon organoids. They were able to demonstrate successful engraftment of these donor organoids through endoscopic monitoring for up to 6 months (Sugimoto et al., 2018). Most notably, the authors determined that the Lgr5^+^ cells were located at the bottom of the crypts of the xenograft, not the mouse crypts. These cells had the ability to migrate up the crypt villus axis 10 days after TAM-induced labeling, and on day 28, the Lgr5^+^ cell progeny cells formed ribbon-like structures (indicative of proliferation up the crypt-villus axis), which indicates that these cells were xenograft derived and had the ability to self-renew. The xenografted Lgr5^+^ cells were able to differentiate into goblet cells, enterocytes, Tuft cells, and EE cells (Sugimoto et al., 2018). In conclusion, these cells have the ability to self-renew and differentiate into fully functioning mature cells of either the secretory or absorptive lineage.

This study was followed by Khalil et al. (2019) looking to replace the damaged murine SI epithelium with either mouse- or human-derived intestinal crypts. The authors injured the jejunal epithelium using EDTA and dithiothreitol (DTT) injection through a 25-gauge needle, followed by gentle scraping of the epithelium. Following injury, GFP^+^ lentiviral-transduced enteroids or spheroids (depending on the shape prior to implantation), were infected into the site. Two distinct surgical models were used: (1) An in-continuity model, which was an implantation of the jejunal segment that was in continuity of the SI, or (2) A bypassed jejunal segment model, which did not receive a regular stream of bowel contents. The authors found that the “in-continuity” model had no engraftments of GFP^+^ labeled murine cells post implant day 7. However, in the bypass model, GFP-expressing enteroids and spheroids were able to successfully engraft 75% (3 of 4 recipients), and demonstrated the ability to fully differentiate, as shown by expression of lysozyme (Paneth cell), Muc2 (goblet cell), synaptophysin (EE cell), and CD10 (small intestinal brush border marker) via immunofluorescence (IF), with engraftment lasting up to 4 weeks. Intestinal transplantation with human cells demonstrated similar differentiation patterns as the aforementioned murine cells, displaying *E*-cadherin, lysozyme, chromogranin A (Paneth cell), and Muc2; however, spheroids showed no expression of differentiation markers, but were Lgr5^+^. In contrast, using human derived organoids, there was limited success of engraftment using the bypass model, which demonstrated a 36% engraftment success of surviving mice (61% survival).

This study provides some evidence of organoid transplantation success, but fails to provide evidence that this system is sustainable for long term engraftment. The reason behind the moderate engraftment rates and low survivability of the mice could be from the DTT treatment, not the EDTA treatment, as Sugimoto and colleagues did not see low survivability rates (Sugimoto et al., 2018). DTT treatment may indeed be altering the epithelial mesenchymal network past the point of successful of engraftment. In order to have successful engraftment, the epithelium cannot be too damaged—it likely needs to have proper signaling, blood flow, and tissue scaffolding to remain intact in order to have successful engraftment into the crypt. These needs were demonstrated recently by Meran et al. (2020), who used intestinal crypts from biopsies from children with intestinal failure/short bowel, to produce functional organoids. Growing these organoids on decellularized human extracellular matrix scaffolds (obtained from separate pediatric intestinal resection tissue) led to jejunal grafts that were able to survive and partially differentiate when implanted into mice.

These studies provide a basis for future strategies using organoid transplantation to ameliorate inflammatory intestinal diseases. By CRISPR-Cas9 gene knock-in or knock-out strategies, one could simultaneously improve efficacy in anti-cytokine therapies by maximizing anti-cytokine targeted receptors, while re-establishing the damaged epithelium with healthy Lgr5^+^ stem cells. Combining cytokine therapy and autologous organoid transplantation could also help reduce the inflammatory environment in the recipient, thus aiding in the integration of organoids into the damaged intestinal epithelium. Moreover, in a chronic setting, one could obtain mucosal biopsies and actively select for a regenerative stem cell population that has the ability to rescue the inflamed epithelium. Further studies could look into the type of growth factors and extracellular matrix that facilitate growth of regenerative cells to help aide in this mechanism. There will be difficulties, however, to obtain and successfully expand autologous ISC-derived enteroids, as well as safely and efficiently clearing the damaged intestinal ISC populations from the patient, to ensure successful engraftment for long term ISC replacement. Future studies will need to focus on how to successfully engraft organoids into the intestinal epithelium, but most importantly, be able to maintain proper signaling to ensure appropriate self-renewal and differentiation into mature epithelial cells, without creating tumorigenic potential.

## Conclusion

The process of self-renewal and rapid turnover that evolved in the intestinal epithelium functions extremely well in most circumstances, rendering most enteric infections and epithelial injuries non-lethal. Even when damage is profound enough to destroy crypts and their CBCs, other cells are able to step in and repair the injury, helping preserve barrier, absorptive, and secretory functions and reducing the potential for long-term harm to the organisms. However, the down side of this process may be propagation of long-lived cellular and epigenetic changes that impair intestinal homeostasis and predispose to IBD, cancer, and other diseases. Recent studies into the cellular processes that underlie crypt repair, including defining specific epithelial and mesenchymal cell types that participate, have great potential to allow for manipulation of these processes to prevent and treat these diseases.

## Author Contributions

WR conceived and wrote majority of the manuscript. RT and EY assisted with research and produced the figures. NZ provided important scientific content and contributed to editing. TS provided financial support, wrote portions of the manuscript, and edited the manuscript and figures. All the authors contributed to the article and approved the submitted version.

## Conflict of Interest

The authors declare that the research was conducted in the absence of any commercial or financial relationships that could be construed as a potential conflict of interest.

## Abbreviations

AA: arachidonic acid
ChIP: chromatin immunoprecipitation
CBCs: crypt-base columnar cells
DARSCs: damage associated regenerative stem cells
DCs: dendritic cells
DSS: dextran sodium sulfate
DTR: diptheria toxin receptor
EC: enterochromaffin
EC: enteroendocrine
EGF: epidermal growth factor
ER: endoplasmic reticulum
HNF4 α: hepatocyte nuclear factor 4 alpha
IBD: inflammatory bowel disease
IECs: intestinal epithelial cells
IF: immunofluorescence
ILC3: group 3 innate lymphoid cells
iPSCs: induced pluripotent stem cells
LP: lamina propria
MSCs: mesenchymal stem cells
revSCs: revival crypt cell populations
RSCs: reserve stem cells
SI: small intestine
TA: transit amplifying cells
TAM: tamoxifen
TSS: transcriptional start sites
UPR: unfolded protein response
IECs: intestinal epithelial cells
ISCs: intestinal stem cells
ChIP: chromatin immunoprecipitation
DCs: dendritic cells
LP: lamina propria
EGF: epidermal growth factor
ER: endoplasmic reticulum
UPR: unfolded protein response
IBD: inflammatory bowel diseases
HNF4a: hepatocyte nuclear factor 4 alpha
CBCs: crypt-base columnar cells
RSCs: reserve stem cells
TA: transit amplifying cells
TSS: transcriptional start sites.
